# Relationship between nonalcoholic fatty liver disease and bone mineral density in elderly Chinese

**DOI:** 10.1186/s13018-023-04168-8

**Published:** 2023-09-13

**Authors:** Guangheng Zhang, Yingsong Zhao, Siyuan Wang, Qing Gong, Hewei Li

**Affiliations:** 1 Department of Orthopaedics, Liyuan Hospital, Tongji Medical College, Huazhong University of Science and Technology, Wuhan, 430077 China; 2 Department of Hand Surgery, Union Hospital, Tongji Medical College, Huazhong University of Science and Technology, Wuhan, 430077 China; 3 Department of Gastroenterology, Tumor Immunology and Cytotherapy, Medical Research Center, The Affiliated Hospital of Qingdao University, No. 1677 Wutaishan Road, Huangdao District, Qingdao, 266000 China

**Keywords:** Osteopenia, Nonalcoholic fatty liver disease, Bone mineral density, Aging population, Cross-sectional study

## Abstract

**Objective:**

As our society grows older, both bone loss and fatty liver have become important issues. However, the relationship between reduced bone mineral density and fatty liver remains controversial. The purpose of this study was to investigate the relationship between nonalcoholic fatty liver disease and less bone mineral density in the ankles of Chinese people over 65.

**Methods:**

The research included 716 older adults over the age of 65 who underwent a comprehensive health screening. A logistic regression model was used to investigate the relationship between nonalcoholic fatty liver disease (NAFLD) and ankle bone mineral density.

**Results:**

A logistic regression model was used to analyze the odds ratios of reduced bone mineral density between patients with fatty liver and controls. In the adjusted model, adjustments were made for age, sex, systolic blood pressure, diastolic blood pressure, overweight rate, history of hypertension, history of diabetes, history of smoking, history of alcohol consumption, fasting glucose, hemoglobin, urea, creatinine, triglycerides, total cholesterol, high-density lipoprotein cholesterol, low-density lipoprotein cholesterol, waist circumference, total protein, albumin, and globulin. The adjusted OR (aOR) for reduced bone mineral density was 0.66 [95% confidence interval (CI) = 0.45–0.97, *P* = 0.034 < 0.05]. In subgroup analysis by age, sex, and BMI, women, age > 75 years, and BIM < 25 were statistically significant.

**Conclusion:**

This study suggested that NAFLD is associated with a reduced risk of reduced heel bone mass. More research needs to be done to back up the results of this study and look into possible problems.

## Introduction

Osteoporosis is a bone metabolic disease that causes a loss in bone density as well as an increased risk of fragility fractures in patients [1]. Relevant epidemiological research indicates that osteoporosis is a pretty common condition in the world that primarily affects the elderly [2]. The overall prevalence of osteoporosis among people over 40 years of age in China is 19.74% and 24.62% [3]. Numerous earlier researches have demonstrated a connection between it and BMI, smoking, drinking, diabetes, dyslipidemia, and systemic chronic inflammatory disorders [4, 5].

Fatty liver is a chronic liver condition caused by an abnormal buildup of fat in the liver cells. It is one of the most frequent liver illnesses in the globe [6, 7]. The prevalence of NAFLD among adults globally ranges from 17 to 46%, with Chinese individuals having the highest prevalence at 29.2%. Over time, fatty liver disease has become more common among people in China. Obesity, type 2 diabetes, and hereditary factors all have a connection to NAFLD [8–10]. It has many characteristics similar to osteoporosis.

In numerous earlier investigations on fatty liver and osteoporosis, an increasing number of studies have demonstrated a correlation between the two conditions [11–15]. However, the findings drawn from the investigation of this relationship are not identical. In the general US population, NAFLD and liver fibrosis are not associated with decreased femoral bone mineral density, according to Stefano Ciardullo [16], and in Hejun Li, it was stated that metabolic-associated fatty liver disease and liver stiffness were associated with higher femoral and lumbar spine BMD in individuals aged 50 and older [17]. NAFLD, however, was linked in a Korean study to risk variables for men's femoral neck bone density and protective factors for women's lumbar spine bone density [13]. Each of these studies mentioned above explored the relationship between bone mineral density and fatty liver in different parts of the body, such as the femur, the femoral neck, and the lumbar spine; however, bone mineral density varies throughout the body [18], and there is a paucity of the literature exploring the relationship between nonalcoholic fatty liver disease and ankle bone mineral density in older adults.

Due to cost, safety, lack of radiation, and simplicity of operation, dual X-rays were not available to the study's senior participants for examination and screening in community hospitals. As mentioned in the literature of some comparative experiments [19], bone ultrasound has a unique advantage in the measurement of bone density at specific sites such as the heel bone, and ultrasound has a high accuracy in the diagnosis of bone density [20–22], and bone ultrasound has been used for the determination of bone density of the heel bone in the research methodology of some literature [23, 24].

The purpose of this study was to review the results of a community health screening to investigate the association between NAFLD and participants' ankle bone mineral density.

## Methods

### Study population

This was a cross-sectional research of 734 healthy persons aged 65 and older who had standard health screening, ultrasound bone mineral density screening, and abdominal ultrasonography at the Yangchunhu Community Health Center in Wuhan, Hubei Province, China, in 2021. Our criteria for inclusion were as follows: (1) age 65 and higher; (2) abdomen and heel bone density ultrasonography with results; and (3) no missing data. Exclusion criteria included a history of hepatitis, anti-osteoporosis medications, and a history of cancer. Based on the above criteria, 716 participants (male, *n* = 305 and female, *n* = 411) were eventually included in this study. The study was approved by the Ethics Committees at Liyuan Hospital, Tongji Medical College, and Huazhong University of Science and Technology, and all participants provided written informed permission.

### Clinical and laboratory assessment

The relevant study's data gatherers were in charge of getting information from the participants, such as their age, height, weight, and measurements of their waist and other body parts. Weight (kg) divided by height squared (m^2^) is the formula used to determine the body mass index (BMI). BMI less than or equal to 25 was regarded as the normal range and higher than or equal to 25 as the overweight range [13]. Relevant history of smoking, alcohol consumption, hypertension, diabetes, medications, and related surgeries was asked through a standardized short questionnaire. By smoking history, participants were divided into two groups: the current smoking/drinking group (current or past smoking/drinking) and the non-smoking/drinking group. The patient rested for 10 minutes before the blood pressure measurement. After an overnight fast, venous blood samples were drawn from the elbow, and laboratory tests included fasting blood sugar, hemoglobin, urea (Urea), creatinine (Cr), uric acid (UA), total cholesterol (TC), triglycerides (TG), high-density lipoprotein cholesterol (HDL-C), low-density lipoprotein cholesterol (LDL-C), alanine aminotransferase (ALT), aspartate aminotransferase (AST), total protein (TP), albumin (Alb), globulin (GLB), and total bilirubin (T-Bil).

### Measurement of bone density and NAFLD

OSTEOKJ3000 ultrasound heel bone densitometry, according to the WHO diagnostic criteria, subjects with a T-score of − 1.0 SD or lower (based on comparison with the mean osteo-sono assessment index score in young adults (20–44 years)) were defined as having reduced bone mass [25, 26]. NAFLD was diagnosed using Mirror2 (Blue Rhythm) based on an abdominal ultrasound. The ultrasound was performed by one of the three liver sonographers with at least 5 years of specialized experience. The diagnosis of fatty liver (hepatic steatosis) was made by ultrasound based on the brightness of the liver, the contrast between the echoes of the liver and the kidneys, the depth of attenuation, and the vascular structure. And the diagnosis of NAFLD was made by asking for personal history to exclude any significant alcohol abuse, history of chronic toxic liver disease, etc.

### Statistical analysis

Using SPSS 24.0 (IBM Corp. Released 2016. IBM SPSS Statistics for Windows, Version 24.0. Armonk, NY: IBM Corp.), all data were analyzed and categorized by fatty liver. In this study, categorical variables were tested using Chi-square tests, and continuous numerical variables were tested using *t*-tests. *P* values of < 0.05 were considered to indicate statistical significance.

The OR of fatty liver in bone loss was investigated using logic regression models. The crude and adjusted models [age, sex, systolic blood pressure, diastolic blood pressure, overweight rate, history of hypertension, history of diabetes, history of smoking, history of alcohol consumption, fasting glucose, hemoglobin, urea, creatinine, triglycerides, total cholesterol, high-density lipoprotein cholesterol, low-density lipoprotein cholesterol, waist circumference, total protein, albumin, and globulin] were used. Then, in subgroups according to age, sex, and obesity rate, the subgroups for age were 75 years as the cutoff.

## Results

### Characteristics of the participants

**Fig. 1:**
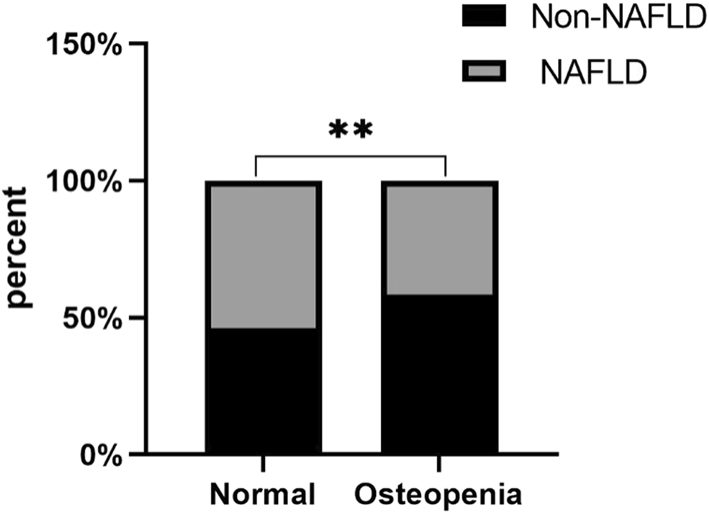
NAFLD presence was the percentage of occurrence of heel bone loss. *P* values were calculated by the Chi-square test. **P < 0.01

The general characteristics and laboratory data of the participants are shown (384 Non-NAFLD, 332 NAFLD) in Table 1. Age, fasting glucose, hemoglobin, uric acid, TC, TG, HDL-C, LDL-C, WC, ALT, AST, TP, Alb, GLB, T-Bil, obesity rate, history of hypertension, history of diabetes mellitus, and heel bone mineral density were significantly different between non-fatty liver and alcoholic fatty liver patients. Figure 1 shows that the osteopenia ratio between the alcoholic fatty liver group and the nonalcoholic fatty liver group was statistically significant.

**Table 1:** Baseline characteristics of the study participants

Characteristics	Non-NAFLD (*n* = 384)	NAFLD (*n* = 332)	*P* value
Age	71.640 ± 6.050	70.610 ± 4.786	**0.011**
SBP (mmHg)	135.350 ± 16.428	136.770 ± 17.581	0.267
DBP (mmHg)	77.530 ± 9.863	78.970 ± 10.519	0.059
FBS (mmol/L)	5.672 ± 1.770	6.366 ± 2.266	** < 0.001**
Hemoglobin (g/L)	136.891 ± 14.331	140.377 ± 13.850	**0.001**
Urea (mmol/L)	5.657 ± 1.597	5.455 ± 1.568	0.089
Cr (μmol/L)	80.289 ± 41.086	76.048 ± 21.089	0.077
UA (μmol/L)	308.448 ± 95.258	342.810 ± 90.804	** < 0.001**
TC (mmol/L)	4.847 ± 1.035	5.023 ± 1.060	**0.025**
TG (mmol/L)	1.360 ± 0.814	1.961 ± 1.173	** < 0.001**
HDL-C (mmol/L)	1.383 ± 0.354	1.192 ± 0.261	** < 0.001**
LDL-C (mmol/L)	2.999 ± 0.893	3.242 ± 0.901	** < 0.001**
WC (cm)	83.945 ± 8.171	90.570 ± 7.939	** < 0.001**
ALT (U/L)	18.563 ± 8.481	24.515 ± 13.817	** < 0.001**
AST (U/L)	22.122 ± 6.420	23.627 ± 8.761	**0.010**
TP (g/L)	74.629 ± 4.285	76.033 ± 4.157	** < 0.001**
Alb (g/L)	44.568 ± 2.718	45.307 ± 2.262	** < 0.001**
GLB (g/L)	30.063 ± 3.779	31.007 ± 5.305	**0.006**
T-Bil (μmol/L)	14.291 ± 6.203	14.693 ± 6.007	**0.038**
Sex (%)			0.260
Male	44.5	40.4	
Female	55.5	59.6	
Obesity (%)			** < 0.001**
No (BMI < 25 kg/m^2^)	81.3	41.0	
Yes (BMI ≥ 25 kg/m^2^)	18.8	59.0	
Hypertension (%)			** < 0.001**
Yes	45.8	59.0	
No	54.2	41.0	
Diabetes (%)			**0.041**
Yes	19.8	26.2	
No	80.2	73.8	
Smoking status (%)			0.298
Yes	16.7	13.9	
No	83.3	86.1	
Drinking status (%)			0.373
Yes	16.1	18.7	
No	83.9	81.3	
Bone density (%)			**0.001**
Normal	34.6	46.4	
Osteopenia	65.4	53.6	

Data presented as mean ± standard deviation or percentage

*P* values according to *t*-test or Chi-square test

Boldface type indicates a significant *p* value (*p* < 0.05)

Non-NAFLD: normal individuals without NAFLD

*NAFLD* nonalcoholic fatty liver disease, *BMI* body mass index, *WC* waist circumference, *DBP* diastolic blood pressure, *SBP* systolic blood pressure, *FPG* fasting plasma glucose, *TC* total cholesterol, *TG* triglycerides, *AST* aspartate aminotransferase, *ALT* alanine aminotransferase, *BMD* bone mineral density, *FBS* fasting blood sugar, *HDL-C* high-density lipoprotein cholesterol, *LDL-C* low-density lipoprotein cholesterol, *Cr* creatinine, *UA* uric acid, *TP* total protein, *Alb* albumin, *GLB* globulin, and *T-Bil* total bilirubin

### Relationship between NAFLD and BMD

Table 2 shows the findings of a multiple logistic regression analysis. The adjusted OR for nonalcoholic fatty liver-induced bone loss was 0.66 (95% CI 0.45–0.97; *P* = 0.034), indicating that nonalcoholic fatty liver is a protective factor for decreased heel bone density. Only among women were the results consistent, with an adjusted OR of 0.52 (95% CI 0.30–0.88, *P* = 0.016).

**Table 2:** Relationship between NAFLD and BMD

Characteristics	Crude	*P* value	Adjusted	*P* value
Total				
Non-NAFLD	1.00		1.00	
NAFLD	0.612 (0.453–0.828)	**0.001**	0.662 (0.452–0.968)	**0.034**
Female				
Non-NAFLD	1.00		1.00	
NAFLD	0.540 (0.361–0.807)	**0.003**	0.515 (0.301–0.882)	**0.016**
Male				
Non-NAFLD	1.00		1.00	
NAFLD	0.708 (0.448–1.120)	0.140	0.815 (0.448–1.481)	0.501

Crude and adjusted odd ratios (95% confidence interval) of NAFLD for osteopenia. Logistic regression model, significance at *P* < 0.05. Models adjusted for age, sex, systolic blood pressure, diastolic blood pressure, overweight rate, history of hypertension, history of diabetes, history of smoking, history of alcohol consumption, fasting glucose, hemoglobin, urea, creatinine, triglycerides, total cholesterol, high-density lipoprotein cholesterol, low-density lipoprotein cholesterol, waist circumference, total protein, albumin, and globulin

### Subgroup analysis

In the other two subgroup analyses, as demonstrated in Table 3, where the subgroup was older than or equal to 75 years, the adjusted OR was 0.33 (95% CI 0.12–0.94, *P* = 0.039); in the non-obese group, the adjusted OR was 0.74 (95% CI 0.34–0.93, *P* = 0.024).

**Table 3:** Relationship between NAFLD and BMD stratified by age and BMI

Characteristics	Crude	*P* value	Adjusted	*P* value
Age < 75				
Non-NAFLD	1.00		1.00	
NAFLD	0.712 (0.508–0.998)	**0.048**	0.755 (0.496–1.150)	0.191
Age ≥ 75				
Non-NAFLD	1.00		1.00	
NAFLD	0.428 (0.210–0.872)	**0.020**	0.330 (0.116–0.944)	**0.039**
BMI < 25				
Non-NAFLD	1.00		1.00	
NAFLD	0.597 (0.395–0.901)	**0.014**	0.559 (0.337–0.928)	**0.024**
BMI ≥ 25				
Non-NAFLD	1.00		1.00	
NAFLD	0.762 (0.441–1.319)	0.332	0.735 (0.392–1.377)	0.336

Crude and adjusted odd ratios (95% confidence interval) of NAFLD for osteopenia. Logistic regression model, significance at *P* < 0.05. Models adjusted for age, sex, systolic blood pressure, diastolic blood pressure, overweight rate, history of hypertension, history of diabetes, history of smoking, history of alcohol consumption, fasting glucose, hemoglobin, urea, creatinine, triglycerides, total cholesterol, high-density lipoprotein cholesterol, low-density lipoprotein cholesterol, waist circumference, total protein, albumin, and globulin

Bold values indicate* p* values < 0.05

## Discussion

NAFLD was found to be an independent protective factor for heel bone mineral density in a study of participants over 65 years of age. This study examined the relationship between NAFLD and bone mineral density on ultrasound. NAFLD continued to be a protective factor for decreased heel bone mineral density in the female subgroup, age > 75 years, and non-obese group.

In the previous studies, the relationship between NAFLD and BMD remains controversial; however, our findings indicate that the OR for bone loss in NAFLD was lower than in controls after controlling for other variables (0.66, 95% CI 0.45–0.97, *P* = 0.034). And this result had a lower OR for reduced bone mineral density in women with NAFLD than in controls (0.52; 95% CI 0.30–0.88; *P* = 0.016). The findings of this paper are consistent with the results of many previous studies that have used DXA to determine BMD at selected study sites to explore the relationship between BMD and NAFLD [13, 17, 27]. However, in some studies using the same research approach, the opposite conclusion was reached that there is a negative correlation between NAFLD and BMD [12, 28]. NAFLD was negatively associated with lumbar spine BMD in a cross-sectional study of 3739 postmenopausal women in Korea [29]. And in another Japanese study on NAFLD and BMD, it was suggested that NAFLD is a risk factor for BMD [11]. Several factors may have contributed to the differences in the results of the studies: first, differences in the locations where BMD was examined; second, differences in the participants themselves, such as sex ratio, age composition, racial distribution, and lifestyle habits; and third, the methods used to measure BMD, such as the use of dual X-rays or bone ultrasound to detect BMD. However, the strength of this research is that it examines the relationship between fatty liver and lumbar spine BMD, femoral neck BMD, and metacarpal bone density in the majority of studies [12, 29]. Few studies have explored the association between heel bone and NAFLD; therefore, this study was designed to examine, at a mean age of > 65 years, the relationship between NAFLD and decreased right heel bone mineral density by evaluating the T-score of the right heel bone.

Numerous prior researches have examined the mechanisms between NAFLD and BMD. However, the pathophysiological link between the two has not been fully explored, and the past research indicates that the association between the two is due to many reasons.

Insulin resistance and chronic inflammation have received more attention in prior research. According to studies on insulin resistance and NAFLD, NAFLD worsens the body's insulin resistance, which, in turn, promotes the production of inflammatory agents, an aberrant bone microenvironment, and vitamin D insufficiency, all of which raise the risk of osteoporosis [30, 31]. In addition to chronic inflammation, NAFLD also belongs to the category of chronic inflammation, where chronic inflammation of the organism causes, for example, imbalance of transforming growth factor-beta (TGF-β), increase in tumor necrosis factor-alpha, alteration of vitamin D metabolism [32, 33], and alteration of osteopontin, osteoprotegerin, osteocalcin, and fetuin-A [30]. Different degrees of influence are exerted by the aforementioned variables on bone metabolism attempts. Although there are many studies on the effect of NAFLD on BMD, there are still many unknown factors and a great deal of research is still required to investigate the mechanisms and to better understand the relationship between the two and apply them more precisely in clinical settings. In the subgroup analysis of our study, the prevalence of NAFLD was 40.4% in males and 59.6% in women, whereas the prevalence of NAFLD was 17–46% in adults worldwide, since the prevalence of NAFLD is higher in men and postmenopausal women, as stated in a prior study [34, 35]. Consequently, in the discussion based on gender as a grouping variable, only the bone mineral density group of women with NAFLD had a significantly lower OR than the control group, and in one study, bone mineral density decreased with age regardless of whether or not the participants had NAFLD [11]. Therefore, we classified them according to age, and our analysis revealed that the OR for bone loss in the NAFLD group was considerably lower in the 75-plus age group than in the control group. In our analysis, however, the obesity rate was much greater in the fatty liver group (59%) than in the control group (18.3%), and obesity is connected not only with cardiovascular and cerebrovascular illnesses but also with the onset of osteoporosis [36]. In the non-obese NALFD group, the adjusted OR was considerably lower than in the control group. This evidence demonstrates that a low BMI is protective against osteoporosis development.

Firstly, the present experiment is a cross-sectional experiment, which makes it difficult to derive a causal relationship between NAFLD and bone loss due to the limitations of the experiment itself; the use of bone ultrasonography as a method of determining BMD in the heel bone may be limiting as DXA is the gold standard; however, the method is inexpensive, portable, and predictive of articular fracture as well as densitometric measurements of the joints; all of which could potentially detract from the present experiment's exploration of the relationship between BMD and NAFLD. Additionally, all of the study subjects in China were older than 65; therefore, the findings may not be generalizable to people in other nations.

## Conclusions

In conclusion, after adjusting for possible confounders, our findings suggested that NAFLD is associated with a reduced risk of bone loss. In subgroup analyses by sex, age, and BMI, statistical significance was observed in older women, in the age > 75 years group, and in the non-obese group. Longitudinal studies are needed to further explore the relationship.

## Data Availability

The data materials in this paper are from the private data of Wuhan Yangchunhu Community Hospital. If the later reviewers need it, I can provide relevant data materials.
